# Guess who’s coming home: insights into *Aedes albopictus* house-entering behaviour in Southern Europe

**DOI:** 10.3389/fpubh.2026.1843997

**Published:** 2026-10-21

**Authors:** Pedro María Alarcón-Elbal, Ignacio Gil-Torró, Marcos López-de-Felipe, María Altagracia Rodríguez-Sosa

**Affiliations:** 1 Vector-Borne Diseases Research Group (ZOOVEC), Department of Animal Production and Health, Public Veterinary Health and Food Science and Technology, Faculty of Veterinary Medicine, Universidad Cardenal Herrera-CEU, CEU Universities, Alfara del Patriarca, Spain; 2 Vector-Borne Diseases Research Group (ZOOVEC), Department of Pharmacy, School of Health Sciences, Universidad Cardenal Herrera-CEU, CEU Universities, Alfara del Patriarca, Spain; 3 Department of Microbiology and Parasitology, Faculty of Pharmacy, Universidad Complutense de Madrid, Madrid, Spain; 4 Athisa Medio Ambiente, Grupo SASTI, Granada, Spain

**Keywords:** Culicidae, endophily, monitoring, mosquito, Spain, sticky trap

## Abstract

**Introduction:**

*Aedes albopictus* is an invasive species that has spread widely across Europe. In addition to being an irritating pest, it poses a significant public health threat due to its ability to transmit various arboviruses. Despite its biology and ecology having been extensively studied, quantitative evidence of its house-entering behaviour is lacking. We aimed to assess the degree of indoor housing occupancy through the first systematic monitoring of the indoor presence of *Ae. albopictus* in Europe.

**Methods:**

Oviposition traps containing sticky cards were set up in the exterior and interior parts of eight houses in a residential area in eastern Spain. A structured questionnaire was administered to the residents to survey their perceptions of the presence of mosquitoes within their households.

**Results:**

The traps mainly captured *Ae. albopictus* (96.7%), and a very limited number of *Culex pipiens* (3.7%). During the study period, 10.9% of *Ae. albopictus* females were found indoors. Temperatures were shown as the most important variable driving mosquito abundance based on a general additive mixed model (GAMM) and successive hierarchical partitioning. Annoyance was reported to be much higher outdoors than indoors.

**Discussion:**

We demonstrated that *Ae. albopictus* consistently entered all sampled houses at a noticeable rate. The observed correlations between captures and seasonal temperature suggest a positive effect, indicating that global warming may increase mosquito indoor activity in the near future. Further research is needed to shed light on some knowledge gaps regarding the main drivers of house-entering behaviour of *Ae. albopictus* and its potential impact on public health.

## 1 Introduction

*Aedes albopictus* (Diptera: Culicidae), commonly known as the Asian tiger mosquito, being native to Southeast Asia, has undergone an extraordinary geographic expansion over the past five decades, becoming one of the most invasive mosquito species worldwide (1). Beyond its well-established role as a vector of dengue, Zika, and chikungunya viruses, *Ae. albopictus* has demonstrated experimental competence for at least 32 additional arboviruses, positioning it—after the yellow fever mosquito, *Aedes aegypti*—as the second most important mosquito species involved in global arbovirus transmission (2).

In Europe, research on *Ae. albopictus* has evolved substantially since its initial detection in Albania in 1979 (3), progressing from studies focused on species identification, distribution, and basic biology to more integrative approaches addressing its ecological impact, vector competence, control strategies and involvement in autochthonous outbreaks of dengue and chikungunya (4). Despite these advances, key aspects of the bioecology of this vector remain unresolved; notably, one of the most persistent gaps in our understanding being the extent of its activity within human infrastructures.

Traditionally regarded as a highly anthropophilic, yet predominantly exophilic and exophagic species that rarely enters human dwellings to feed or breed (5), accumulating evidence suggests that this assumption may be overly simplistic. In this regard, Delatte et al. (6) documented in Réunion (a tropical French territory in the Indian Ocean) that 11.1% of mosquitoes attempting to bite a human volunteer were captured indoors, while 12.3% of resting mosquitoes were collected inside houses, albeit using sampling methods that differed between environments. Similiar studies conducted in Brazil reported that between 7.2 and 9.8% of *Ae. albopictus* females were collected indoors using human landing catches (7). In Asia, Jin et al. (8) demonstrated indoor oviposition activity in an apartment building in China, with positive ovitraps recorded even on the eighth floor. In Europe, however, evidence remains extremely scarce and limited to a single case report describing indoor breeding and winter persistence of this invasive mosquito species in Germany (9).

This study represents the first systematic assessment of the indoor activity of the Asian tiger mosquito in Europe, aiming to provide a deeper understanding of its tendency to enter human dwellings. By deploying passive traps to collect *Ae. albopictus* both indoors and outdoors, we sought to confirm and quantify at a pilot scale the indoor presence throughout the mosquito season in a highly infested residential area of eastern Spain, while also examining the influence of seasonal phenology and climatic factors, including extreme temperatures, on this behavioural pattern.

## 2 Materials and methods

### 2.1 Study area and mosquito sampling

The study was conducted from early April—recording the first seasonal emergence of adult *Ae. albopictus*—to December 2025 in the Montesol urbanisation, a residential area within the municipality of La Eliana (province of Valencia, eastern Spain). Montesol constitutes a typical peri-urban Mediterranean setting, predominantly composed of detached single-family houses with private gardens. Adjacent peri-domestic public spaces comprised vacant plots consisting of Mediterranean shrubland and low vegetation, including carob trees (*Ceratonia siliqua*), Aleppo pines (*Pinus halepensis*), olive trees (*Olea europaea*), and mastic shrubs (*Pistacia lentiscus*), along with other less abundant native Mediterranean plant species. Eight detached residential houses within a 500 radius were selected for the study. All the houses were characterised by typical suburban features, including private gardens, trees, ornamental vegetation, and swimming pools. The selection of the study site was based on the representativeness of this type of residential areas among the Mediterranean bassin, the homogeneity of the houses in terms of size and structure, and the confirmed sustained presence of *Ae. albopictus* acknowledged through comments from residents and previous published research (10). Household selection responded to the willingness of residents to participate in the research.

Prior to the start of the study, the residents reported being aware of domestic mosquito control measures for the species; however, the research team provided no interventions nor guidance during the study period and did not inspect nor modify potential household breeding sites as to not cause an experimental bias. Participants were also asked to maintain their usual practices, which could affect the entry of mosquitoes into houses.

Adult mosquitoes were sampled using the INESTRAP® (Inesfly Corporation S. L., Paiporta, Spain), an oviposition sticky trap (ST) specifically designed to attract and capture gravid female *Aedes* mosquitoes seeking an oviposition site. A previous field study conducted with this trap demonstrated its effectiveness and capture profile (10). The trap consists of a black plastic container filled exclusively with water, without any chemical or biological attractants, and covered with a mesh to prevent mosquito oviposition. Two sticky cards are installed to capture adult mosquitoes entering the trap, mostly females seeking an oviposition site. Within each studied residence a ST was placed both outside and inside. Outdoor-STs were placed in the gardens of participants, near vegetation, and sheltered from rainfall and direct sunlight exposure. Indoor-STs were set in the living room. All traps were positioned at ground level and remained at the same points throughout the entire study period. Sticky cards were inspected and replaced on a fortnightly basis. The water inside the trap was also renewed after each inspection, and the adhesive strips were transported to the laboratory for analysis as specified by the manufacturer. Adult mosquitoes adhered to the strips were examined under a stereomicroscope. All specimens were sexed and subsequently identified to species level using the identification keys of Schaffner et al. (11). All identifications were performed by the same investigator. The resulting data were used to assess species composition, sex, and temporal capture pattern in both intra- and peri-domestic environments.

### 2.2 Climatic data

The area is characterized by mid-to-high temperatures recorded throughout the year. Relative humidity is consistently high, and precipitation is largely absent during the hot summer months, and abundant in autumn (September to November), with occasional torrential storms (>200 mm) (12).

Site specific climatic data from 1 January to 31 December 2025 were obtained directly from the official records of the Valencian Meteorology Association “Josep Peinado” (AVAMET), corresponding to La Eliana weather station, located approximately 1 km from the study area. Daily variables included minimum, maximum and/or mean temperature (°C), relative humidity (%), wind speed (km/h), atmospheric pressure (atm) and precipitation (mm). Daily climatic data were grouped according to monitoring dates using the ‘=AVERAGEIFS()’ formula from Microsoft Excel. Minimum, maximum or mean values for each studied climatic variable were obtained for each sample period.

### 2.3 Participating residents survey

A 16-question survey was created using Google Forms and distributed to the adult head of each of the sampled households at the end of the study. The questionnaire covered topics related to mosquito reports either indoor or outdoor households, female biting behaviour, and the implemented mosquito-proofing elements present in each residence. Surveys included pictures of adult *Ae. albopictus* as a visual reference for participants to exclude potential misidentifications with other present mosquito species. The survey is included as Supplementary Appendix 1.

### 2.4 Data processing, curation, and analysis

Data processing, curation and inferential and descriptive analyses were conducted in Microsoft Excel (Microsoft Office Professional Plus 2021, v. 2,602 for Windows; Microsoft Office) and RStudio (R version 4.3.3. for Windows, The R Foundation for Statistical Computing), while data were represented in GraphPad Prism (GraphPad Software, v. 8.0.2. 263 for Windows). Capture rates per species and sex were compared between indoor and outdoor traps through a non-parametric paired Wilcoxon test, and are represented as a table, bar-plots and pie-charts. Traps were paired by household. The phenological activity of female *Ae. albopictus* captures indoors and outdoors was also represented as a line plot, while the correlation between populations was assessed through a Spearman correlation test. GAMMs were prepared and evaluated for global female and male *Ae. albopictus* population, as well as specifically for indoor and outdoor populations using the ‘gam()’ function from the mgcv package and the ‘summary()’ and ‘AIC()’ functions from baseR. The effect of independent variables within models was addressed using the ‘gam.hp.()’ function from the package “gam.hp.” Climatic and environmental variables were used as fixed effectors, households as a random effector and the number of captured *Ae. albopictus* females was set as the described variable. Minimum and maximum limits for different climatic variables were also evaluated by grouping capture data by reported temperatures and conducting a general linear model (lm) with the “lm()” function from base R. Evaluated variables and parameters of each model are represented as a table in the Supplementary Appendices 2–4. Statistical differences were considered when *p ≤* 0.05, while *p ≤* 0.10 was seen as slightly significant. Model data fitness was assessed through the obtained deviance explained, R^2^, GCV and AIC values.

### 2.5 Ethics statement

All volunteers were informed about the aims of the study before sampling began. Participation was voluntary and without financial compensation, and participants could withdraw at any time without consequences. No confidential information was disclosed, and the questionnaires did not collect any personally identifiable data. To prevent bias, statistical analyses were performed by an analyst blinded to the household location.

## 3 Results

### 3.1 Indoor vs outdoor *Ae. albopictus* captures

A total of 2,425 *Ae. albopictus* (2,063 females and 362 males) and 84 *Culex pipiens* (49 females and 35 males) were captured during the study. To a lesser extent, the trap captured very few *Culiseta longiareolata* and other insects from the families Sciaridae and Psychodidae, which were not included in the analyses. Whilst outdoor captures were significantly higher in all the study groups (Figure 1A), 10.9% of female *Ae. albopictus* were caught indoors (Figure 1B), although a noticeable variation was recorded among studied households (1.41–26.13% of female *Ae. albopictus* captured indoors) (Supplementary Appendix 4). Female *Ae. albopictus* outdoor populations appeared to show an activity plateau in May and October. Similarly, indoor activity was reported from May to September, although most of the captures were restricted to a peak between late July and early August (Figure 1C). Additionally, outdoor and indoor female *Ae. albopictus* populations showed a positive monotonic association population (Spearman test; *ρ* = 0.51; *p* < 0.0001), while also being moderately correlated (General linear model; R^2^ = 0.20; *p* < 0.0001) (Figure 2D).

**Figure 1:**
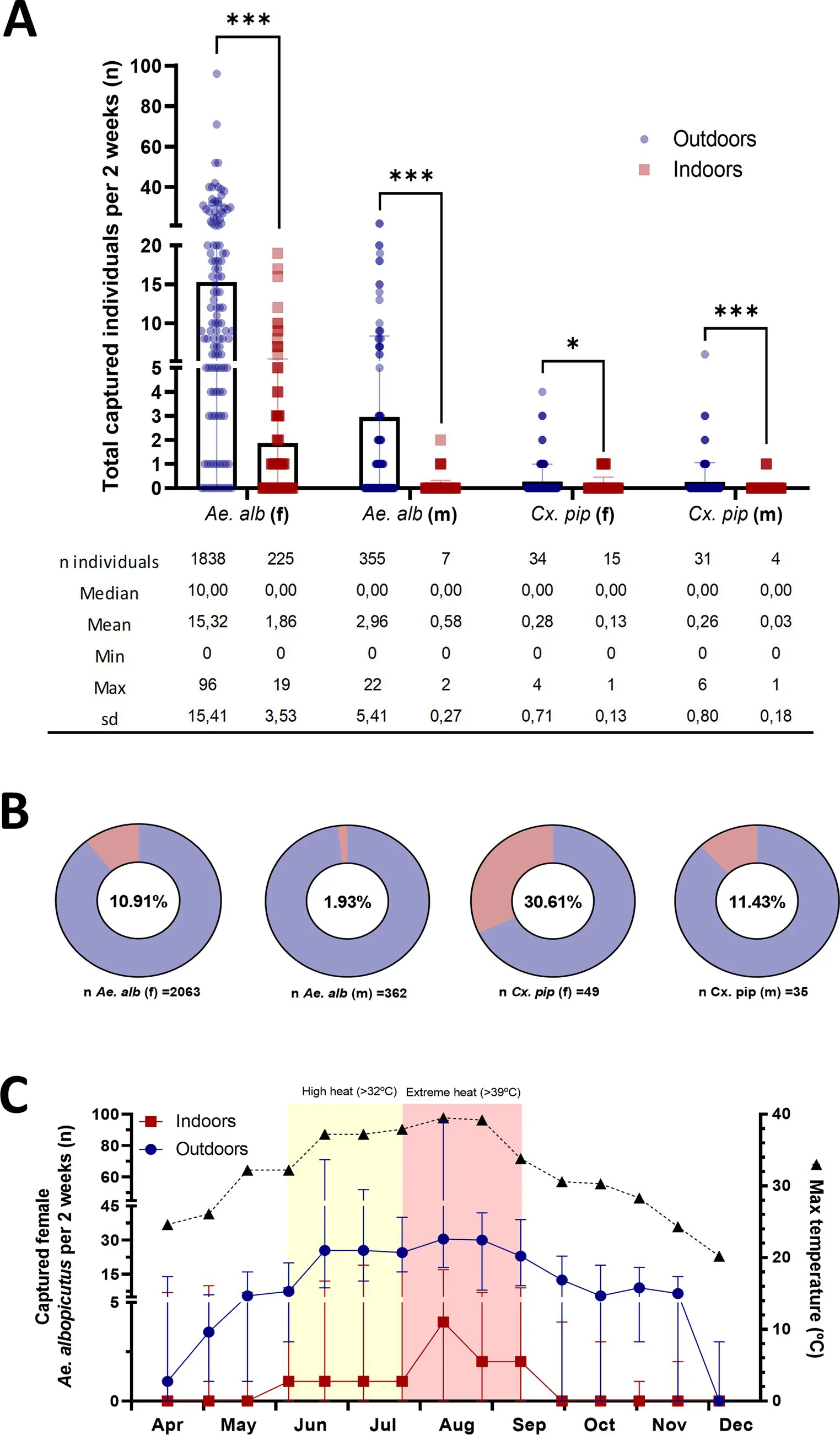
*Aedes albopictus* and other mosquitoes indoor and outdoor activity in a residential area of Valencia. **(A)**. Comparison of outdoor and indoor mosquitoes capture rate. Each point of the bar-diagram represents the number of individuals caught per 2 weeks in traps set either outdoors (blue points) or indoors (red squares). Data was grouped per species (*Ae. alb*.: *Ae. albopictus*; *Cx. pip*.: *Cx. pipiens*) and sex (f: female; m: male) and outdoor and indoor values were compared through a non-parametric paired Wilcoxon test (*p < 0.05; ***p < 0.0001). Bars represent the mean and SD number of captured mosquitoes. Descriptive values are shown as a supplementary table below the graph. **(B)** Outdoor (blue filling) and indoor (red filling) pie-chart of captured individuals per species and sex. Percentage of indoor captured individuals is shown within each chart. **(C)** Outdoor and indoor *Ae. albopictus* female phenological activity and maximum temperature recorded. Each point represents the median value with 95% CI of captured *Ae. albopictus* females per 2 weeks in traps set either outdoors (blue points) or indoors (red squares).

**Figure 2:**
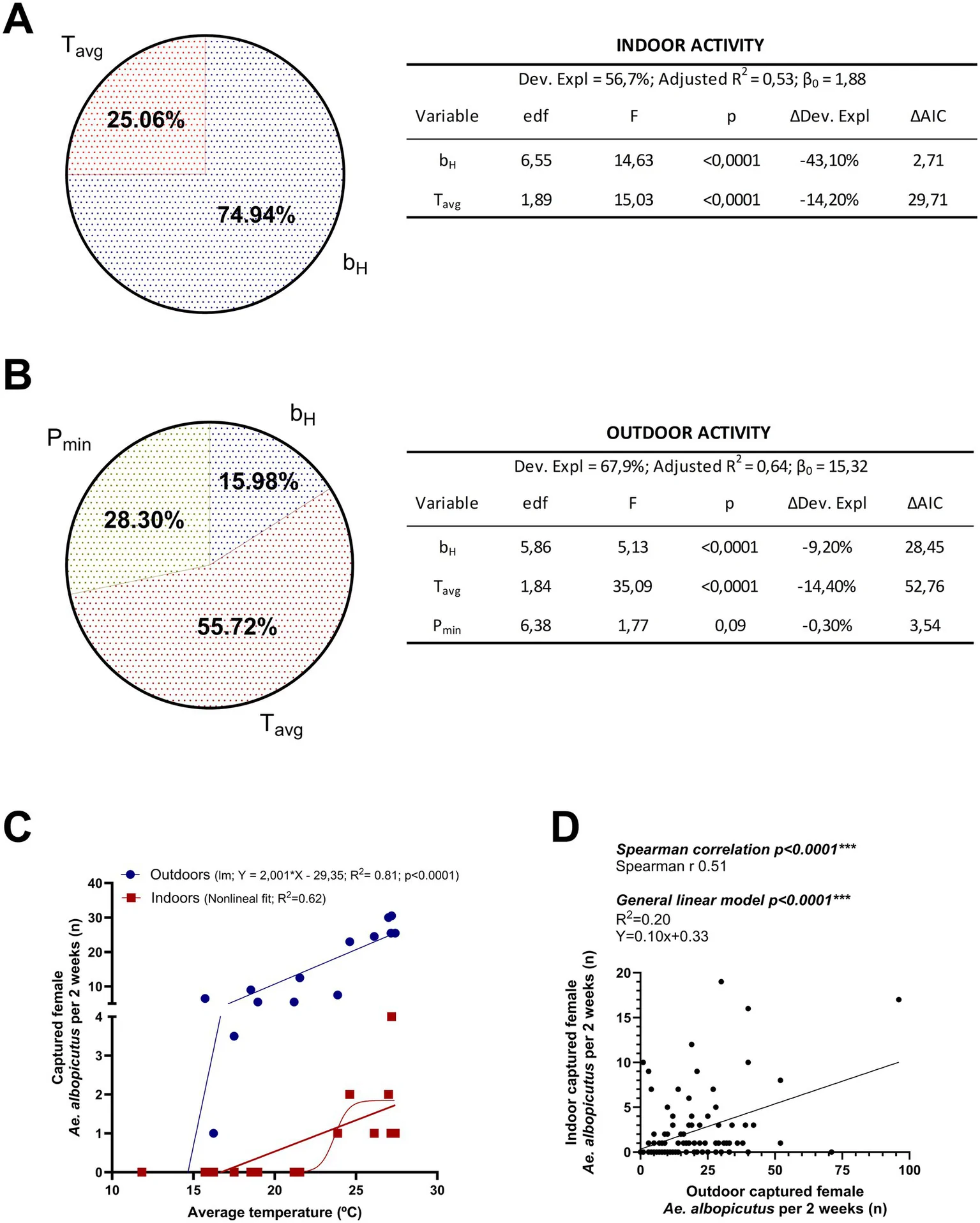
Evaluation of variables associated with female *Ae. albopictus* activity in indoor and outdoor environments. **(A,B)** GAMMs of indoor **(A)** and outdoor **(B)** activity. Pie-charts represent the percentage of representation through hierarchical partitioning of included variables. The results of each model are presented as tables. T_avg_, smooth effect of average weekly temperature; b_H_, random effect of household trapping site; P_min_, weekly average of mean atmospheric preasure; β_0_, intercept, Δ Dev. Expl, variation of deviance explained when variable is excluded; and ΔAIC, variation of AIC predictor when variable is excluded. **(C)** Evaluation of the effect of smoothed average temperatures (X-axis) over median female *Ae. albopictus* capture rate per trap and day (Y-axis) for outdoor (blue dots) and indoor (red squares) populations. Lineal and non-linear fits are presented for outdoor and indoor, respectively. **(D)** Correlation of outdoor and indoor capture rates per trap and study sampling period (lm). Spearman correlation test and general lineal model parameters are also presented.

Anecdotally, during a visit to one of the studied households, a mosquito breeding site was discovered in the water pooled in a plant pot while replacing the indoor sticky cards. Ten larvae were subsequently identified as *Ae. albopictus*.

### 3.2 Effect of household and climatic variables on indoor/outdoor *Ae. albopictus* abundance

Minimum and maximum temperatures showed a threshold effect over mosquito populations. Outdoor mosquito activity was generally restricted (median abundance >0) to min temperatures above 3.4 °C to 7.6 °C, while capture rate was positively correlated with maximum (lm, *β* = 1.68 ± 0.20; adjusted-R^2^ = 0.86, *p* < 0.0001) and minimum temperature (lm, *β* = 1.84 ± 0.27; adjusted-R^2^ = 0.78, *p* < 0.0001). The highest reported temperature during the study period was 39.5 °C. In contrast, indoor activity was restricted to minimum exterior temperatures higher than 14.4–16.6 °C and maximum exterior temperatures above 30.6–32.2 °C. Indoor activity was also positively correlated with maximum (lm, *β* = 0.15 ± 0.04; adjusted-R^2^ = 0.54, *p* < 0.005) and minimum temperatures (lm, *β* = 0.15 ± 0.05; adjusted-R^2^ = 0.43, *p* < 0.01) above the described thresholds.

Based on the deviance explained (Dev. Expl), AIC and adjusted R^2^ values, GAMMs were prepared for both indoor (Dev. Expl = 56.7%; adjusted R^2^ = 0.53) (Figure 2A),

$$N_{i}=\beta_{0}+f(T_{\mathrm{avg}})+b_{H}$$

and outdoor (Dev. Expl = 68.9%; adjusted R^2^ = 0.64) (Figure 2B) female *Ae. albopictus* populations,

$$N_{i}=\beta_{0}+f(T_{\mathrm{avg}})+f(P_{\min})+b_{H}$$

where N_i_ represents the number of daily captured female *Ae. albopictus* per trap based on the intercept (β_0_), the smooth effect of average temperature (T_avg_) and atmospheric pressure (P_min_), as well as the random effect of trapping household (b_H_). The model parameters for the different tested combinations of variables for both indoor and outdoor populations are presented as Supplementary Appendices 2, 3, respectively. Average temperatures were observed to be non-linearly and positively correlated with both mosquito populations (edf_indoors_ = 1.89; edf_outdoors_ = 1.84) (Figure 2C). Additionally, based on hierarchical partitioning, T_avg_ explained most of the observed variance in outdoor populations (55.72%) and some of the indoor mosquito activity (25.06%). While the random effect of household trapping site explained most of the observed variance of indoor activity (74.94%), its effect was much more restricted for outdoor populations (15.98%). Minimum atmospheric pressure values were also observed to explain part of the explained variance (28.30%) (Figures 2A,B). Similar patterns were observed in male individuals, although with a reduced sample size. Variables and indicators of male outdoor and indoor GAMMs, as well as a descriptive representation of external temperatures over indoor and outdoor populations, are presented as Supplementary Appendices 5–7, respectively. R code for conducted GAMMs is presented as Supplementary Appendix 8.

### 3.3 Household owners survey

Based on the results of the surveys, most participants reported the presence of *Ae. albopictus* indoors (71.4%) and outdoors (100%). Similarly, 50% of respondents reported indoor bites, compared to 100% for exterior parts of the house. Regarding mosquito-proof elements of houses, 87.5% of interviewees said they had screens on some or all windows, while 62.5% stated that they had door protection. The use of insecticides against flying insects was outlined to be low outdoors (12.5%) and notably higher indoors (37.5%). Mosquito annoyance was rated at a mean score of 8.1 outdoors and 3.4 indoors (max. 10).

## 4 Discussion

Our study provides the first systematic, season-long quantification of *Ae. albopictus* presence inside human dwellings in a representative European setting with a Mediterranean temperate climate, demonstrating that a consistent proportion of females were detected indoors throughout the mosquito season in eastern Spain. *Aedes albopictus* has traditionally been regarded as primarily inhabiting peri-domestic environments, in contrast to the general indoor behaviour of *Ae. aegypti* (13). Nonetheless, our results show that 10.9% of captured females were collected inside houses, challenging this prevailing paradigm. Similar values of indoor activity rate have previously been reported in other ecological contexts and through different capture methods (between 7.2 and 12.3% indoor *Ae. albopictus* activity) (6, 7). Furthermore, the incidental detection of an indoor breeding site in one of the visited households, and the reported indoor biting activity during the conducted surveys both point to a potentially noticeable level of endophilic and endophagic activity by *Ae. albopictus* under certain circumstances. The obtained results show that house entry is not an occasional event, but rather a recurrent behavioural pattern that appears to be affected by unidentified variations in household structures, as well as positively correlated with average temperatures.

The presented data provides a highly resolved characterization of *Ae. albopictus* seasonal phenology through a ST methodology from its initial emergence in mid-April through sustained activity until near the end of the year. A similar activity timeframe was observed by Osorio et al. (14) in southern Portugal, through egg monitoring and adult captures with BG-Sentinel traps. While outdoor populations presented a maximum activity plateau between June and August, indoor activity was more restricted to a peak during August. Both populations were observed to increase under similar conditions (Spearman test; ρ = 0.51; *p* < 0.0001), although not in a similar proportion (General linear model; R^2^ = 0.20; *p* < 0.0001). These results may imply a certain degree of independence among indoor and outdoor populations and/or the existence of factors driving mosquito house-entry. Additionally, the sex ratio (females/males) of individuals captured indoors (32.1) was notably higher than the ratio outdoors (5.2), further supporting the differences between indoor and outdoor populations. The main reasons for their presence within houses remain uncertain due to the variety of stimuli that the ST provides to mosquitoes, albeit shelter seeking during periods of extreme temperatures is considered as a potential explanation. Although this oviposition trap provides visual and olfactory cues that may attract female mosquitoes, its placement indoors, in the living room, combined with the absence of chemical or biological attractants in the water, is likely to generate stimuli of insufficient intensity to be perceived by mosquitoes remaining outdoors. Consequently, its capture efficiency is expected to be limited primarily to individuals that have already entered the house and it is not regarded as a confounding factor.

In this sense, the use of INESTRAP for capturing *Ae. albopictus* is supported by its high efficiency and ecological suitability to *Aedes* oviposition-seeking behaviour. Recent comparative studies conducted in the same geographical area reported significantly higher capture rates for INESTRAP than for BG-GAT, with 2.46–4.34 times more mosquitoes collected, including a greater absolute number of gravid females. Additionally, its low cost, independence from electricity, and easy maintenance make it well-suited for sustained synanthropic *Aedes* surveillance programs (10). However, in the present study, the gonotrophic status of captured females was not assessed, as sticky cards were replaced on a fortnightly basis rather than every three days as in the previously mentioned study; this extended exposure time, particularly under the high temperatures recorded during some months (>39 °C), likely compromised specimen condition and hindered reliable visual determination.

In any case, our data suggests the major importance of temperature over mosquito dynamics in residential environments. Global warming is impacting the biology of mosquitoes, affecting their ability to colonise temperate zones and extending their seasonality (15). Among the consequences of global warming, heat waves have been shown to affect *Ae. albopictus* densities in a complex and location-specific manner. Based on our results, hierarchical partitioning within conducted GAMMs (Figures 2A,B) identified the major positive importance of average weekly temperatures over outdoor mosquito population, while also affecting — to a lower degree — indoor activity, which was mostly explained by household variability. Nonetheless, maximum temperatures were also shown to potentially increase indoor activity, as median mosquito presence within human dwellings was only observed at maximum temperatures higher than 30.6–32.2 °C. Conducted male GAMMs, whilst limited due to the low sample size, also point to a positive association between extreme temperatures and male indoor activity. In this regard, it could be argued that extreme temperature events (Figure 1C) could be an important driver to indoor mosquito activity fostering behavioural plasticity and increasing the contact between vector and human reservoirs, posing major epidemiological implications in arbovirus endemic areas. However, other factors may be involved in this phenomenon, and there is lack of data for a proper comparison.

The factors associated with household variability—the most important variable associated with indoor activity during the conducted model—remain unclear, despite the survey analysis being based mainly on permanent mosquito-proofing measures as a direct proxy for the probability of mosquito entry. Indoor temperature was considered a relevant factor potentially associated with mosquito density and, consequently, as a potential source of variability contributing to uncertainty in the factors driving mosquito entry. Slight differences in outdoor temperature among the eight household gardens were expected and could have influenced mosquito captures and their interpretation in the statistical models, representing an additional limitation of the study.

No correlation was observed between mosquito populations and annoyance, nor preventive measures (mosquito-netting or indoor insecticide applications) (data not shown). Such lack of correlation is believed to be due to the limited sample size (*n* = 8). Larger-scale testing could lead to more robust results in the future. Although only eight houses were monitored, the phenomenon of mosquito entry was consistently recorded in all studied households during the mosquito season, with a mean positivity index of 21.1% from June to September, although much variation was observed among houses (1.41–26.13%). Additionally, while the present work was not designed to assess indoor breeding, and no systematic search for intra-domiciliary larval habitats was conducted, the aforementioned discovery of a breeding site with ten *Ae. albopictus* larvae inside one of the surveyed homes suggests that indoor oviposition may occur under suitable conditions. This observation should therefore be interpreted cautiously, as it does not demonstrate regular indoor colonisation but rather indicates a behaviour that may be occasional and context dependent. Importantly, previous research conducted in a similar and geographically close Mediterranean residential area in the province of Valencia (approximately 20 km away) focused exclusively on peri-domestic inspections and did not include a systematic exploration of indoor spaces for breeding sites (16). By contrast, studies that have actively surveyed indoor environments, such as those conducted in the Dominican Republic (17), have generally reported little to no evidence of *Ae. albopictus* breeding sites within households, suggesting that indoor colonisation is likely infrequent, although it may vary in different epidemiological scenarios. However, in this Caribbean context, the high abundance of *Ae. aegypti*—a species strongly adapted to indoor environments and absent from mainland Spain—may act as a key competitive pressure, potentially limiting the establishment and oviposition of *Ae. albopictus* within households.

Regarding *Cx. pipiens*, a mosquito species of significant public health relevance, collections revealed that over 30% of females (*n* = 15) were captured indoors, indicating a notable level of endophilic activity. In this sense, the total number of *Ae. albopictus* recorded indoors was noticeable, reflecting its strong propensity to exploit domestic environments. The predominance of the Asian tiger mosquito in our collections, relative to the traditionally domestic *Cx. pipiens*, may suggest a partial shift of the latter toward more extra-domestic habitats, potentially driven by interspecific interactions and habitat displacement following the establishment of this invasive species. Indeed, growing evidence supports interactions between these two species, with data from southern Spain (18) indicating a preference of *Cx. pipiens* for peri-urban and rural environments. However, uncertainty remains regarding the suitability of this ST for capturing *Culex* individuals (10) and the extent to which the study area accurately reflects the surrounding urbanisation gradient.

*Aedes albopictus* continues to expand its geographic range more than two decades after its initial detection in Catalonia in 2004 (19). Recent entomological surveillance has documented new introductions in certain Spanish regions, including Galicia (20) and the Canary Islands (21). Despite its long-term presence in Spain, fundamental aspects of *Ae. albopictus* bioecology remain poorly characterised. Given the significant public health threat posed by this competent arbovirus vector, it is imperative to further elucidate vector–host interactions within domestic environments. Based on our preliminary findings, the impact of heat waves during outbreaks should also be considered as a potential risk factor, as elevated temperatures were observed to increase indoor mosquito activity and potentially increase human-mosquito contact. Traditional approaches targeting peri-domestic breeding sites and adult collections may underestimate the epidemiological relevance of indoor *Ae. albopictus* activity, particularly considering the extent of indoor entry documented in our study. Future research should extend this finding in other representative European settings, resolving critical knowledge gaps related to mosquito house-entry, especially within the current context of global change, including the gonotrophic status of females and household-specific factors influencing indoor resting and oviposition behaviour, to enhance surveillance and vector control strategies.

## Data Availability

The raw data supporting the conclusions of this article will be made available by the authors, without undue reservation.
