# Supplementary material for: Guess who’s coming home: insights into Aedes albopictus house-entering behaviour in Southern Europe
Source: Front Public Health. 2026 Oct 21;14:1843997. doi: 10.3389/fpubh.2026.1843997 (PMC13731263; doi:10.3389/fpubh.2026.1843997)
Supplement: Supplementary file 1 [file Supplementary_file_1.pdf]

Supplementary Material

Supplementary Appendix 1. Survey results

Question	Results
Did you observe mosquitoes INSIDE your home during the study period?	
Yes	62.5%
No	37.5%
If so, can you confirm that they were tiger mosquitoes?	
Yes	71.4%
No	28.6%
Did you observe mosquitoes outside your home during the study period?	
Yes	100%
No	0%
If so, can you confirm that they were tiger mosquitoes?	
Yes	100%
No	0%
Have you been bitten by mosquitoes inside your home during the study period?	
Yes	50%
No	50%
If so, at what time(s) of day did these bites occur?	
Day	50%
Night	0%
Both	50%
If so, can you confirm that you were specifically bitten by tiger mosquitoes inside your home?	

Yes<br>No	50%<br>50%
Have you been bitten by mosquitoes outside your home during the study period?<br>Yes<br>No<br>If so, at what time(s) of day did these bites occur?<br>Day<br>Night<br>Both<br>If so, can you confirm that you were specifically bitten by tiger mosquitoes outside your home?<br>Yes<br>No	 100%<br>0%<br><br>25%<br>12.5%<br>62.5%<br><br>75%<br>25%
Does your house have mosquito nets on the windows?<br>Yes, in all of them<br>Yes, in some of them<br>No	 62.5%<br>25%<br>12.5%
Does your house have mosquito nets or curtains on the exterior doors?<br>Yes, in all of them<br>Yes, in some of them<br>No	 37.5%<br>25%<br>37.5%
Do you regularly use insecticide products for flying insects inside your house?<br>Yes<br>No	 37.5%<br>62.5%

Do you regularly use insecticide products for flying insects outside your house?	
Yes	12.5%
No	87.5%
How would you rate the annoyance of mosquitoes inside your home (where 0 is no annoyance and 10 is unbearable annoyance)?	3.4
How would you rate the annoyance of mosquitoes outside your home (where 0 is no annoyance and 10 is unbearable annoyance)?	8.1

Supplementary Appendix 2. Evaluation parameters of tested models for female *Aedes albopictus* outdoor activity. ID, model id; b_H, random effect caused by household trapping site; T_{avg}, smoothed effect (se) of weekly average temperatures (ºC); T_{max}, se of weekly maximum temperature (ºC); T_{min} se of weekly minimum temperature (ºC); Hr_{avg}, se of weekly average relative humidity (%); Hr_{max}, se of weekly maximum relative humidity (%); W_{avg}, se of weekly average wind speed (km/h); W_{max}, se of weekly maximum wind speed (km/h); $PREC_{avg}$, se of weekly average precipitations (mm/m^2); P_{avg}, se of weekly average atmospheric pressure (bar); Pmax, se of weekly maximum atmospheric pressure (bar); P_{min}, se of weekly minimum atmospheric pressure (bar); Dev. Expl, deviance explained; ΔDev. Expl, variation of deviance explained (evaluated model Dev. Expl – selected model Dev. Expl), ΔAIC, variation of AIC (evaluated model AIC – selected model AIC). **, p<0.005; ***, p<0.0001; ns, non-significant effect (significance of smoothed terms). Grey shading, selected model.

ID	bH	Tavg	Tmax	Tmin	Hravg	Hrmax	Wavg	Wmax	PRECavg	Pavg	Pmax	Pmin	Dev. Expl	ΔDev. Expl	R^2	AIC	ΔAIC
1	**												42,5%	-14,2%	0,392	592,364	30,074
2	***	***											56,7%	0,0%	0,534	562,29	0
3	***	ns	ns										57,2%	0,5%	0,534	563,24	0,95
4	***	ns		ns									56,7%	0,0%	0,53	564,26	1,97
5	***	***			ns								56,7%	0,0%	0,532	563,27	0,98
6	***	***				ns							57,1%	0,4%	0,534	563,22	0,93
7	***	***					ns						56,7%	0,0%	0,531	563,59	1,3
8	***	***						ns					57,0%	0,3%	0,533	563,47	1,18
9	***	***							ns				56,7%	0,0%	0,531	563,62	1,33
10	***	***								ns			56,7%	0,0%	0,53	564,21	1,92
11	***	***									ns		56,9%	0,2%	0,532	563,72	1,43
12	***	***										ns	56,7%	0,0%	0,53	564,25	1,96

Supplementary Appendix 3. Evaluation parameters of tested models for female *Aedes albopictus* indoor activity. ID, model id; b_H, random effect caused by household trapping site; T_{avg}, smoothed effect (se) of weekly average temperatures (ºC); T_{max}, se of weekly maximum temperature (ºC); T_{min} se of weekly minimum temperature (ºC); Hr_{avg}, se of weekly average relative humidity (%); Hr_{max}, se of weekly maximum relative humidity (%); W_{avg}, se of weekly average wind speed (km/h); W_{max}, se of weekly maximum wind speed (km/h); $PREC_{avg}$, se of weekly average precipitations (mm/m^2); P_{avg}, se of weekly average atmospheric pressure (bar); Pmax, se of weekly maximum atmospheric pressure (bar); P_{min}, se of weekly minimum atmospheric pressure (bar); Dev. Expl, deviance explained; ΔDev. Expl, variation of deviance explained (evaluated model Dev. Expl – selected model Dev. Expl), ΔAIC, variation of AIC (evaluated model AIC – selected model AIC). **, p<0.005; ***, p<0.0001; ns, non-significant effect (significance of smoothed terms). Grey shading, selected model.

ID	bH	Tavg	Tmax	Tmin	Hravg	Hrmax	Wavg	Wmax	PRECavg	Pavg	Pmax	Pmin	Dev. Expl	ΔDev. Expl	R²	AIC	ΔAIC
1	*												10,9%	-57,0%	0,08	994,41	102,73
2	***	***											63,7%	-4,2%	0,61	895,22	3,54
3	***	ns	ns										64,3%	-3,6%	0,613	894,98	3,3
4	***	**		ns									64,1%	-3,8%	0,61	896,05	4,37
5	***	***			ns								64,0%	-3,9%	0,609	896,29	4,61
6	***	***				ns							66,9%	-1,0%	0,625	895,22	3,54
7	***	***					ns						64,2%	-3,7%	0,61	896,21	4,53
8	***	***						ns					63,8%	-4,1%	0,606	897,13	5,45
9	***	***							ns				63,8%	-4,1%	0,907	897,04	5,36
10	***	***								ns			64,1%	-3,8%	0,609	896,32	4,64
11	***	***									ns		64,1%	-3,8%	0,61	896,17	4,49
12	***	***										*	67,9%	0,0%	0,64	891,68	0

Supplementary Appendix 4. Comparison of indoor and outdoor female *Aedes albopictus* activity per household (Figure 1). Each point represents the total of captured individuals per trap and trapping period for outdoor (blue dots) and indoors (red squares). Data was grouped per studied household. A paired Wilcoxon test was conducted per group (*, p<0.05).

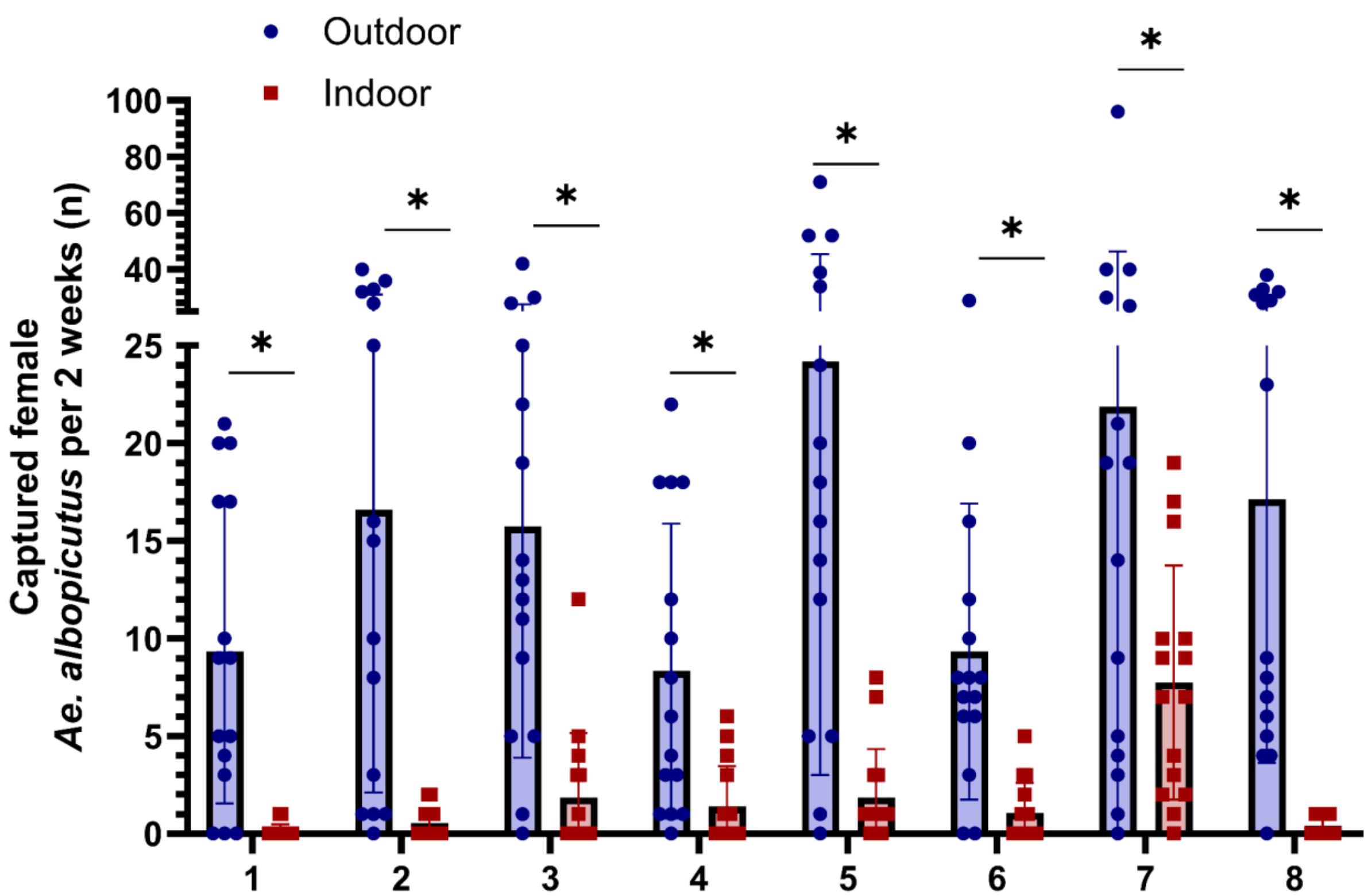

	1	2	3	4	5	6	7	8
Outdoor	98,59%	96,89%	89,39%	85,62%	92,84%	89,74%	73,87%	97,72%
Indoor	1,41%	3,11%	10,61%	14,38%	7,16%	10,26%	26,13%	2,28%

Supplementary Appendix 5. Evaluation parameters of tested models for male *Ae. albopictus* outdoor activity. ID, model id; b_H, random effect caused by household trapping site; T_{avg}, smoothed effect (se) of weekly average temperatures (ºC); T_{max}, se of weekly maximum temperature (ºC); T_{min} se of weekly minimum temperature (ºC); Hr_{avg}, se of weekly average relative humidity (%); Hr_{max}, se of weekly maximum relative humidity (%); W_{avg}, se of weekly average wind speed (km/h); W_{max}, se of weekly maximum wind speed (km/h); $PREC_{avg}$, se of weekly average precipitations (mm/m^2); P_{avg}, se of weekly average atmospheric pressure (bar); Pmax, se of weekly maximum atmospheric pressure (bar); P_{min}, se of weekly minimum atmospheric pressure (bar); Dev. Expl, deviance explained; ΔDev. Expl, variation of deviance explained (evaluated model Dev. Expl – selected model Dev. Expl), ΔAIC, variation of AIC (evaluated model AIC – selected model AIC). **, p<0.005; ***, p<0.0001; ns, non-significant effect (significance of smoothed terms). Grey shading, selected model.

ID	bH	Tavg	Tmax	Tmin	Hravg	Hrmax	Wavg	Wmax	PRECavg	Pavg	Pmax	Pmin	Dev. Expl	ΔDev. Expl	R^2	AIC	ΔAIC
1	ns												<0,01 %	<0,01 %	<0,01	28,14	8,163
2	ns	**											12,3%	-11,5%	0,0997	18,52	-1,457
3	ns	ns	ns										13,7%	-10,1%	0,103	19,576	-0,401
4	ns	ns		ns									12,3%	-11,5%	0,0922	20,48	0,503
5	ns	*			ns								19,6%	-4,2%	0,137	18,358	-1,619
6	ns	**				ns							11,9%	-11,9%	0,0912	20,037	0,06
7	ns	**					ns						23,2%	-0,6%	0,161	16,643	-3,334
8	ns	**						ns					14,3%	-9,5%	0,11	18,38	-1,597
9	ns	*							ns				12,1%	-11,7%	0,092	20,286	0,309
10	ns	**								0,08			23,8%	0,0%	0,174	19,977	0
11	ns	*									ns		22,5%	-1,3%	0,156	17,141	-2,836
12	ns	***										ns	18,3%	-5,5%	0,134	17,329	-2,648

Supplementary Appendix 6. Evaluation parameters of tested models for male *Aedes albopictus* indoor activity. ID, model id; b_H, random effect caused by household trapping site; T_{avg}, smoothed effect (se) of weekly average temperatures (ºC); T_{max}, se of weekly maximum temperature (ºC); T_{min} se of weekly minimum temperature (ºC); Hr_{avg}, se of weekly average relative humidity (%); Hr_{max}, se of weekly maximum relative humidity (%); W_{avg}, se of weekly average wind speed (km/h); W_{max}, se of weekly maximum wind speed (km/h); $PREC_{avg}$, se of weekly average precipitations (mm/m^2); P_{avg}, se of weekly average atmospheric pressure (bar); Pmax, se of weekly maximum atmospheric pressure (bar); P_{min}, se of weekly minimum atmospheric pressure (bar); Dev. Expl, deviance explained; ΔDev. Expl, variation of deviance explained (evaluated model Dev. Expl – selected model Dev. Expl), ΔAIC, variation of AIC (evaluated model AIC – selected model AIC). **, p<0.005; ***, p<0.0001; ns, non-significant effect (significance of smoothed terms). Grey shading, selected model.

ID	bH	Tavg	Tmax	Tmin	Hravg	Hrmax	Wavg	Wmax	PRECavg	Pavg	Pmax	Pmin	Dev. Expl	ΔDev. Expl	R^2	AIC	ΔAIC
1	**												17,4%	-35,5%	0,137	735,802	60,208
2	***	***											52,9%	0,0%	0,492	675,594	0
3	***	**	ns										53,6%	0,7%	0,496	675,31	-0,284
4	***	*		ns									53,1%	0,2%	0,489	677,195	1,601
5	***	***			ns								52,9%	0,0%	0,488	677,43	1,836
6	***	***				ns							53,2%	0,3%	0,49	677,221	1,627
7	***	***					ns						53,5%	0,6%	0,494	675,9845	0,3905
8	***	***						ns					53,2%	0,3%	0,491	676,748	1,154
9	***	***							ns				52,9%	0,0%	0,488	677,336	1,742
10	***	***								ns			53,8%	0,9%	0,497	675,439	-0,155
11	***	***									ns		53,3%	0,4%	0,4914	676,739	1,145
12	***	***										ns	55,7%	2,8%	0,505	675,98	0,386

Supplementary Appendix 7. Time evolution of *Aedes albopictus* male captures outdoor and indoor (Figure 2). Outdoor and indoor *Ae. albopictus* male phenological activity and maximum temperature recorded. Each point represents the median value with 95% CI of captured *Ae. albopictus* males per 2 weeks in traps set either outdoors (blue points) or indoors (red squares).

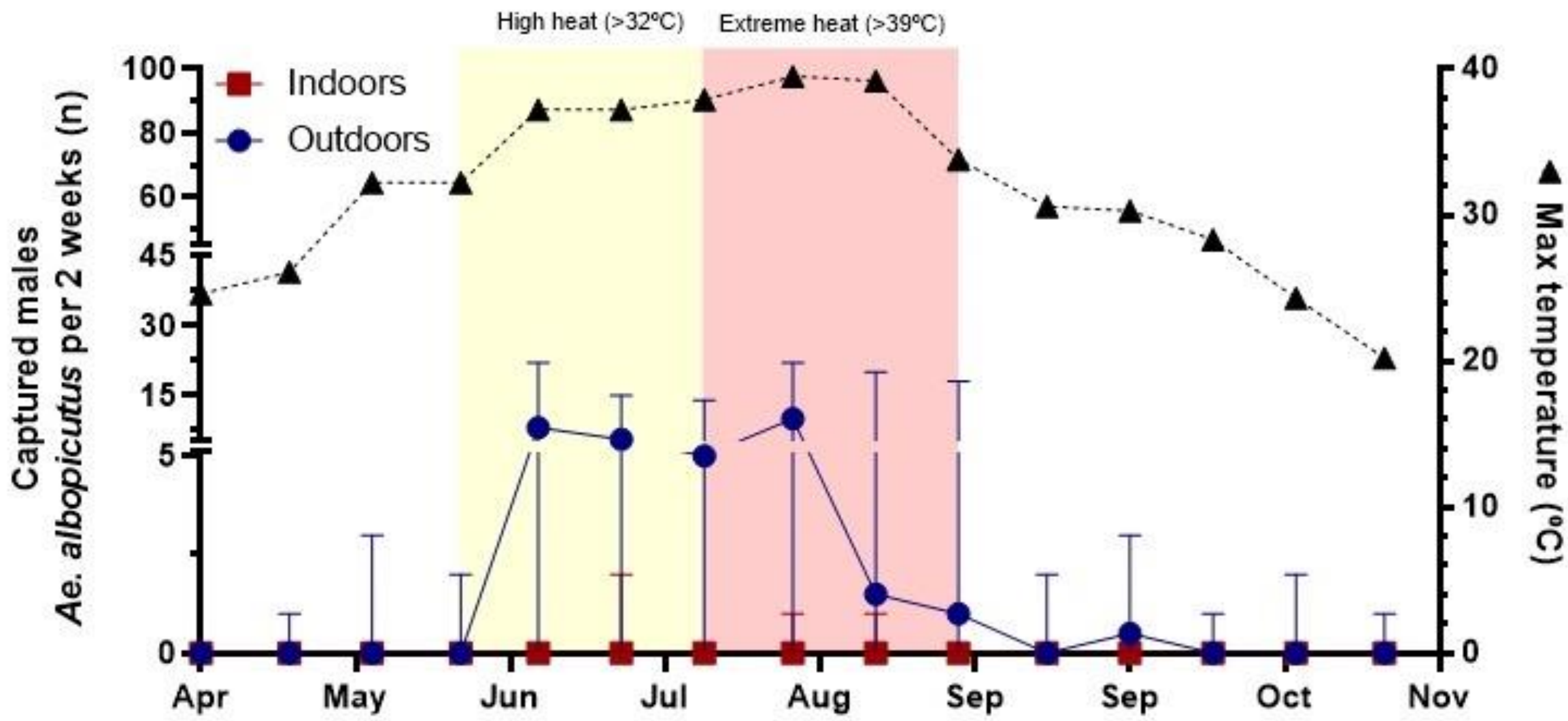

Supplementary Appendix 8. GAMM R code accessibility. Example of code for outdoor female activity. Indoor female and both indoor and male populations were modeled similarly.

```
#Loading libraries

library(readxl)

library(mgcv)

library(dplyr)

library(ggplot2)

library(gam.hp)

library(writexl)


#Preparing data.frame

INESTRAP$HOUSE <- as.factor(INESTRAP$HOUSE)

INESTRAP$SITE <- as.factor(INESTRAP$SITE)

INESTRAP$SP <-as.factor(INESTRAP$SP)

INESTRAP$SEX <- as.factor(INESTRAP$SEX)
```

```
INESTRAP2<- INESTRAP %>% mutate(DATE=as.numeric(DATE_initial))


#INDOOR_POP
INESTRAP2<- INESTRAP %>% mutate(DATE=as.numeric(DATE_initial))%>%
  filter(SITE=="OUTSIDE")%>% filter(SP=="Ae. albopictus")%>%
  filter(SEX=="female")


#model1: phenology and date per sp and sex
model1<- gam(N~s(DATE)+s(HOUSE, bs="re"),
        data=INESTRAP2, family=gaussian())
summary(model1)
AIC(model1)
#model2: temp med
model2<- gam(N~s(DATE)+s(HOUSE, bs="re")+s(TEMP_X),
        data=INESTRAP2, family=gaussian())
summary(model2)
AIC(model2)


#model3: temp max
model3<- gam(N~s(DATE)+s(HOUSE, bs="re")+s(TEMP_X)+s(TEMP_MAX),
        data=INESTRAP2, family=gaussian())
summary(model3)
AIC(model3)


#model4: temp min
```

```
model4<- gam(N~s(DATE)+s(HOUSE, bs="re")+s(TEMP_X)+s(TEMP_MIN),
        data=INESTRAP2, family=gaussian())
summary(model4)
AIC(model4)

#model5: HRmed
model5<- gam(N~s(DATE)+s(HOUSE, bs="re")+s(TEMP_X)+s(HR_X),
        data=INESTRAP2, family=gaussian())
summary(model5)
AIC(model5)

#model6: HR_min
model6<- gam(N~s(DATE)+s(HOUSE, bs="re")+s(TEMP_X)+s(HR_MIN),
        data=INESTRAP2, family=gaussian())
summary(model6)
AIC(model6)

#model7: WIND_MED
model7<- gam(N~s(DATE)+s(HOUSE, bs="re")+s(TEMP_X)+s(WIND_X),
        data=INESTRAP2, family=gaussian())
summary(model7)
AIC(model7)

#model8: WIND_MAX
model8<- gam(N~s(DATE)+s(HOUSE, bs="re")+s(TEMP_X)+s(WIND_MAX),
        data=INESTRAP2, family=gaussian())
```

```
summary(model8)
AIC(model8)

#model9: PREC_M
model9<- gam(N~s(DATE)+s(HOUSE, bs="re")+s(TEMP_X)+s(PREC_M),
        data=INESTRAP2, family=gaussian())
summary(model9)
AIC(model9)

#model10: ATM_X
model10<- gam(N~s(DATE)+s(HOUSE, bs="re")+s(TEMP_X)+s(ATM_X),
        data=INESTRAP2, family=gaussian())
summary(model10)
AIC(model10)

#model11: ATM_MAX
model11<- gam(N~s(DATE)+s(HOUSE, bs="re")+s(TEMP_X)+s(ATM_MAX),
        data=INESTRAP2, family=gaussian())
summary(model11)
AIC(model11)

#model12: ATM_MIN
model12<- gam(N~s(DATE)+s(HOUSE, bs="re")+s(TEMP_X)+s(ATM_MIN)+s(HR_X),
        data=INESTRAP2, family=gaussian())
summary(model12)
```

```
AIC(model12)


#model13: temp max NO MED
model13<- gam(N~s(DATE)+s(HOUSE, bs="re")+s(TEMP_MAX),
            data=INESTRAP2, family=gaussian())
summary(model13)
AIC(model13)


#model14: temp min
model14<- gam(N~s(HOUSE, bs="re")+s(TEMP_X),
            data=INESTRAP2, family=gaussian())
summary(model14)
AIC(model14)



model14b<- gam(N~s(TEMP_X),
             data=INESTRAP2, family=gaussian())
summary(model14b)
AIC(model14b)



model14c<- gam(N~s(HOUSE, bs="re"),
             data=INESTRAP2, family=gaussian())
summary(model14c)
AIC(model14c)
```

```
#Lineal representation
plot(model14)
ggplot(INESTRAP2, aes(x=TEMP_X, y=N)) + geom_point() + geom_smooth()

hp.indoor <- gam.hp(model14, type="adjR2")
hp.indoor
```
